# Outcome of distal femoral replacement: Influence of trauma vs. elective indications – A prospective single-center study

**DOI:** 10.1371/journal.pone.0354235

**Published:** 2026-07-24

**Authors:** Anne Elisabeth Postler, Franziska Beyer, Cornelia Lützner, Jörg Lützner

**Affiliations:** University Center of Orthopaedic, Trauma and Plastic Surgery, University Hospital, TU Dresden, Dresden, Germany; Carol Davila University of Medicine and Pharmacy: Universitatea de Medicina si Farmacie Carol Davila din Bucuresti, ROMANIA

## Abstract

**Background:**

Distal femoral replacement (DFR) is often the last leg-saving option in advanced femoral bone defects after total knee arthroplasty (TKA) and periprosthetic distal femoral fractures. The aim of this study was to compare the midterm outcome of DFR following periprosthetic fracture or elective TKA revision.

**Methods:**

This prospective cohort study included 93 DFR patients (49 trauma, 44 elective) from 2010 to 2021. DFR for revision in periprosthetic joint infection (PJI) or tumor resections were not included. Data on demographics, surgical history and reason of failure until DFR, revision rates, satisfaction and pain in mean 5 years postoperatively were collected. Revision incidence was estimated using cumulative incidence functions accounting for death as a competing event, while patient mortality was analysed using Kaplan-Meier estimation. Multivariate Cox regression models were used to assess which parameters were associated with DFR revision.

**Results:**

Trauma patients had in mean 1.5 (SD 0.9) prior surgeries, while elective patients had 3.1 (SD 1.5), p < 0.001. Satisfaction was higher after trauma (6.9/10) compared to elective DFR (5.4/10). The 1- and 5-year cumulative incidence of revision was 4.1% and 13.9% in trauma patients and 7.0% and 42.0% in elective patients, respectively. The 1- and 5-years mortality rates were 18.7% and 47.5% (trauma) and 6.9% and 16.8% (elective). Female sex was associated with lower, previous constraint TKA, and elective indication were associated with higher revision risk.

**Conclusion:**

There were higher revision rates but lower mortality in elective DFR compared to trauma patients, mainly caused by pre-existing conditions. Surgeons should take this into consideration during shared decision making for DFR.

**Registry and the registration number of the study:**

German Clinical Trial Register (DRKS).

DRKS-ID: DRKS00036378.

## Introduction

The number of revision total knee arthroplasty (TKA) is expected to increase by nearly 90% by 2050 in Germany [1]. The most common reasons for revision in tertiary care hospitals are infection (36.1%), aseptic loosening (21.9%) and periprosthetic fracture (13.7%). In cases of severe bone defects and periprosthetic distal femur fractures where internal fixation is not an option, distal femoral replacement (DFR) is considered as salvage procedure to preserve the leg. Nonunion after fixation, reported in up to 24% after distal femur fractures [2], can also necessitate DFR. Long-term survivorship, but high complication rates in DFR are well-known for limb salvage after bone tumor resections in oncological patients [3,4]. While DFR is essential in aseptic revisions with insufficient metaphyseal bone stock, there is ongoing debate about its use versus fixation in fracture patients [5]. Managing these complex cases, especially in elderly patients with osteoporotic bone and comminuted fractures, is challenging and requires individualized decision-making. These geriatric patients need early mobilization [2,5–9]. DFR offers immediate full weight-bearing and avoids the risk of nonunion, making it an appealing option. However, similar complication and revision rates are reported for both, open reduction and internal fixation (ORIF) and DFR, in recently published meta-analysis as well as register-based studies [5,10–13].

This study aimed to compare the revision and mortality rates of DFR performed as a salvage procedure following periprosthetic fracture versus elective revision TKA in a high-volume arthroplasty-center.

## Materials and methods

The institutional arthroplasty registry for revision knee arthroplasty procedures was approved by the Ethics Committee of the University Medicine Carl Gustav Carus, TU Dresden in 2009 (EK348112009). Patients provided written informed consent for inclusion in the registry and prospective documentation of their clinical data.

An amendment for long-term follow-up evaluation of these patients, was approved by the same ethics committee in 2011 (EK288082011). All procedures were conducted in accordance with the Declaration of Helsinki.

This study included all patients who received DFR between 01/2010 and 12/2021 at a university-based tertiary care hospital. Patients who had DFR due to septic revision surgery or tumor resection were excluded. A total of 93 patients were included: (1) 49 trauma patients with periprosthetic distal femoral fractures and no good option for internal fixation (Rorabeck III) [14] and (2) 44 elective patients with aseptic loosening and advanced femoral bone defects (AORI F3) [15]. All distal femoral replacements were performed by senior arthroplasty surgeons from the same specialized arthroplasty unit to ensure consistency of surgical technique. A standardized medial parapatellar approach was used in all cases. Any bone preparation was conducted according to the manufacturer’s recommendations. All components were cemented using vacuum-mixed polymethylmethacrylate (PMMA) with standardized pressurization technique and waiting time to achieve optimal fixation. In all cases, antibiotic-loaded bone cement was applied. Postoperative care followed an institutional protocol including immediate full weight-bearing and daily physiotherapy under supervision to both trauma and elective DFR patients. All patients were mobilized on the first postoperative day with full weight-bearing as tolerated. Daily physiotherapy focused on gait training and gradual range-of-motion exercises, with additional occupational therapy and use of a knee brace or continuous passive motion device if required. Discharge to home or inpatient rehabilitation occurred once safe ambulation and pain control were achieved.

**Fig 1:**
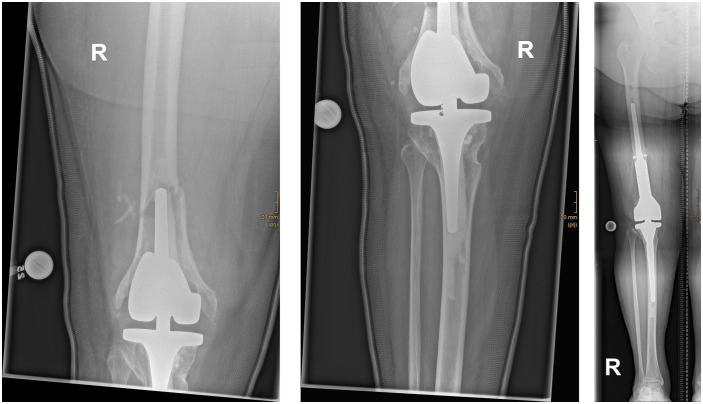
Example radiographs of a trauma patient: (a) preoperative distal femur, (b) preoperative proximal tibia, and (c) postoperative radiograph following distal femoral replacement.

**Fig 2:**
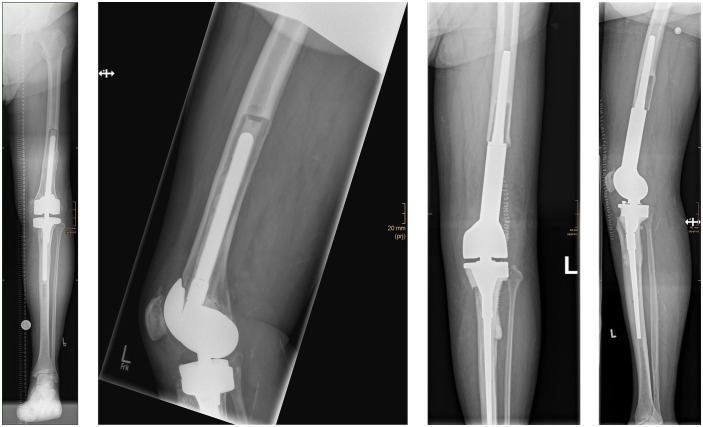
Example radiographs of an elective patient: (a) preoperative full-length anteroposterior view, (b) preoperative lateral view, (c) postoperative anteroposterior view, and (d) postoperative lateral view following distal femoral replacement.

All patients underwent standardized pre- and postoperative imaging according to our institutional arthroplasty protocol, see Figs 1 and 2. Preoperatively, full-length anteroposterior radiographs of the entire lower limb including calibration marker (ball) were obtained to assess mechanical alignment, fracture morphology, and bone loss. Additional lateral and patella views were taken in all cases, and computed tomography (CT) was performed to evaluate bone defects and remaining cortical support. Postoperatively, standardized full-length anteroposterior, lateral, and patella view radiographs were obtained before discharge and at each follow-up visit to assess implant position, alignment, fixation, and radiolucent lines.

Demographic data such as age, sex, Body Mass Index (BMI) and comorbidities (ASA, American Society of Anaesthesiologists classification) [16] were collected. The number of revision surgeries prior to DFR, reasons for failure, kind of explant until DFR, and any revisions and mortality after DFR were recorded. To assess potential selection bias in functional follow-up, baseline characteristics of responders (n = 43) and non-responders (n = 50) were compared.

**Fig 3:**
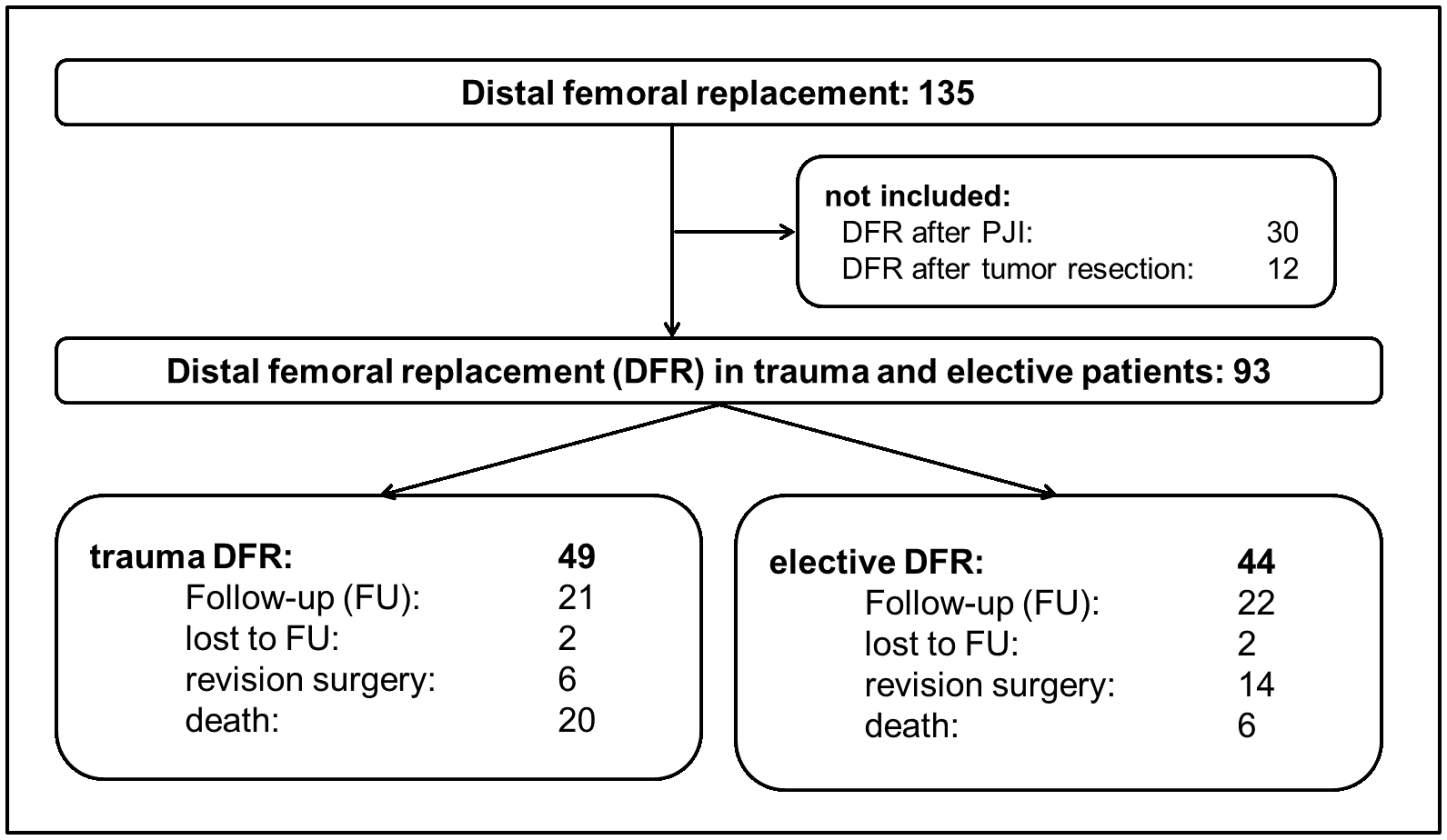
Flowchart of the included patients with distal femoral replacement.

Revision was defined as any surgery involving exchange or removal of any bone-anchored (femoral or tibial) prosthetic component. Constraint described the degree of mechanical coupling in the prior total knee arthroplasty. Cases were grouped into (1) bicondylar (posterior-stabilized or cruciate-retaining) TKAs, (2) semi-constrained or rotating-hinge TKAs providing additional varus–valgus stability, and (3) megaprosthetic constructs with segmental bone replacement. A total of 43 patients (21 trauma DFR patients and 22 elective DFR patients) were available for the follow-up, see Fig 3. Revision DFR were seen in 20 patients, 26 patients died and 4 were lost to follow-up.

At a mean follow-up (FU) of five years (1–10 years), patients completed a questionnaire on satisfaction and pain. Satisfaction and pain were assessed using a 10-point visual analogue scale (VAS) ranging from 0 to 10, where 0 indicated *no satisfaction* or *no pain*, and 10 indicated *maximum satisfaction* or *worst imaginable pain*. Patients rated their current level of satisfaction with the surgical outcome and their average pain during daily activities at the time of follow-up. The questionnaire included further questions on walking distance (not possible, at home, < 500 m, 500–1000 m, > 1km, unlimited), use of walking aids (none, one, two, walker/ wheel chair) and ability to climb stairs (not possible, upstairs normally and downstairs not possible, up- and downstairs using handrails, normally).

### Statistics

Data description was based on means, standard deviation (SD) and ranges for continuous values and absolute and relative frequency for categorial values. To assess potential survivor bias, baseline characteristics of patients were compared with those without (responders vs. non-responders). Differences between groups were analysed using Mann Whitney U test for not normally distributed continuous values and chi-square test for categorical values. Multivariate Cox regression models were used to identify parameters associated with DFR revision. Variables with a p-value < 0.20 in univariable analysis were entered into the multivariable Cox regression model. This screening threshold was chosen to avoid exclusion of potentially relevant predictors and is consistent with common practice in exploratory clinical studies. The final model was adjusted for these variables, and results are presented as hazard ratios (HR) with 95% confidence intervals derived from Cox regression models. The multivariable Cox regression model was based on 20 revision events. Given the limited number of events relative to the number of predictors, the multivariable analysis should be interpreted as exploratory. The cumulative incidence of implant revision was estimated using cumulative incidence functions (CIF) with death treated as a competing event, and group differences were assessed using Gray’s test. Patient mortality was analysed using Kaplan–Meier estimation.

Missing data were not imputed; analyses were performed as complete-case analyses. We compared baseline characteristics between patients with and without available patient-reported outcomes to assess potential response bias.

A p-value of less than 0.05 was considered statistically significant. Data analyses were performed using SPSS (release 28.0 for Windows).

The sample size estimation for revision rates was based on data from previously published studies on distal femoral replacement (DFR). Reported midterm revision rates range from approximately 10–15% for trauma-related DFR and 30–35% for elective revision DFR. Assuming expected 5-year revision rates of 13% in the trauma group and 33% in the elective group, a two-sided comparison of independent proportions (α = 0.05, power = 0.80) would require 76 patients per group (total = 152) to detect a statistically significant difference.

All distal femoral replacements performed since 2010 were documented prospectively in our institutional arthroplasty registry as part of routine clinical quality monitoring. All patients who had undergone surgery in 2010 provided a second written informed consent for participation in the long-term follow-up study. Hence, the design represents an ambispective cohort (retrospective identification of index procedures, prospective outcome collection).

## Results

**Fig 4:**
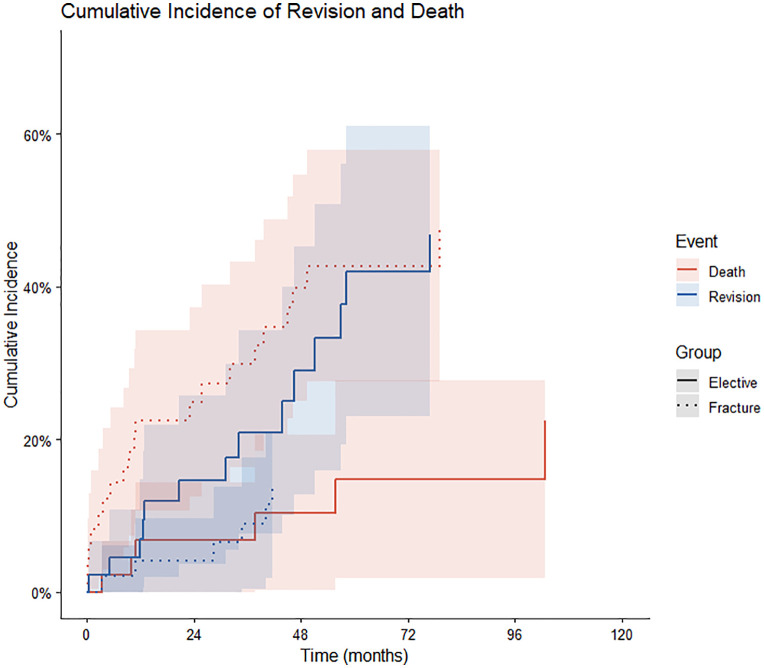
Cumulative Incidence of Revision and Death (blue = revision, red = death).

Baseline characteristics of responders (n = 43) and non-responders (n = 50) were compared to assess potential response bias, see Table 1. The two groups were largely comparable regarding age, sex, ASA classification, and indication (trauma vs. elective), see also Table 2. Trauma patients had a mean of 1.5 (SD 0.9) prior surgeries versus 3.1 (SD 1.5) in elective DFR (p < 0.001). The 1- and 5-year cumulative incidence of revision was 4.1% (95% CI: 1.3–6.9%) and 13.9% (95% CI: 3.3–24.5%) in trauma patients, and 7.0% (95% CI: 0.3–13.7%) and 42.0% (95% CI: 23.0–61.0%) in elective patients, respectively. Group differences in cumulative incidence were statistically significant for both revision (Gray’s test, p = 0.010) and death (Gray’s test, p = 0.005). The corresponding 95% confidence intervals are displayed as shaded areas in Fig 4.

**Table 1:** Comparison of demographic data between responders and non-responders among 93 DFR patients.

	Responders (n = 43)	Non-Responders (n = 50)	
	n (%)	n (%)	p-value
female, n [%]	32 (74.4)	30 (60.0)	.141
ASA grade, n [%]			
1–2	15 (34.9)	14 (28.0)	.475
3–4	28 (65.1)	36 (72.0)	
trauma DFR, n [%]	21 (48.8)	28 (56.0)	.490
elective DFR, n [%]	22 (51.2)	22 (44.0)	
	**mean (SD)**	**mean (SD)**	**p-value**
age at DFR [years]	74.21 (±11.32)	76.34 (±10.01)	.3171
BMI [kg/m²]	30.71 (± 5.83)	29,55 (± 6.05)	.423
lenghth of stay [days]	14.63 (± 7.53)	18.9 (± 14.15)	.440
cut-sew-time [min]	173.67 (± 56.76)	162.50 (± 63.29)	.261
sugergies until DFR [number]	2.37 (± 1.46)	2.20 (± 1.41)	.524

**Table 2:** Comparison of demographic data among 93 DFR patients, differentiated by indication (trauma vs. elective DFR).

	trauma DFR (n = 49)	elective DFR (n = 44)		FU trauma DFR (n = 21)	FU elective DFR (n = 22)	
	n (%)	n (%)	p-value	n (%)	n (%)	p-value
female, n [%]	36 (73.5)	26 (59.1)	0.142	17 (81)	15 (68.2)	0.337
ASA grade, n [%]						
1–2	14 (28.6)	15 (34.1)	0.566	6 (28.6)	9 (40.9)	0.396
3–4	35 (71.4)	29 (65.9)		15 (71.4)	13 (59.1)	
	**mean (SD)**	**mean**	**p-value**	**mean (SD)**	**mean (SD)**	**p-value**
age at DFR [years]	78.5 (11.6)	72.0 (8.4)	0.000	76.7 (14.28)	71.9 (7.1)	0.031
BMI [kg/m²]	29.4 (6.1)	30.8 (5.8)	0.363	29.1 (4.6)	32.2 (6.6)	0.144
lenghth of stay [days]	18.7 (13.2)	13.9 (9.1)	0.000	15.4 (6.9)	13.9 (8.2)	0.097
cut-sew-time [min]	160.0 (56.2)	176.3 (64.1)	0.225	172.9 (56.8)	174.5 (58.1)	0.942
sugergies until DFR [number]	1.5 (0.9)	3.1 (1.5)	0.000	1.6 (1.1)	3,1 (1.4)	0.000
pain [NRS]	5.5 (3.3)	5.0 (2.6)	0.621	5.5 (3.3)	4.9 (2.6)	0.621

Multivariate regression analysis identified previous constraint TKA (vs. bicondylar TKA) as factors associated with higher revision risk, whereas female sex (vs. male) was associated with lower revision risk, see Table 3. Elective indication was associated with a significantly higher risk of revision compared with trauma DFR, whereas age, BMI, numbers of previous surgeries were not independently associated with revision risk, see Table 3. Trauma patients had longer implant survival until DFR (mean 10.5 years; range 0.6–24.6 years) compared to elective patients (mean 4.5 years; range 0.4–18.2 years), p < 0.001. For 40 trauma patients (81.6%), DFR was the first revision surgery, whereas for 81.8% (n = 36) of elective patients, DFR was at least the second revision surgery, p < 0.001. The implants used were comparable, with a similar combined length of the femoral component and stem: 120.5 mm (SD 61) in trauma patients and 125.3 mm (SD 41.6) in elective patients. Reason for failure in all 49 trauma patients were periprosthetic fractures Rorabeck type III. Causes for elective DFR were aseptic loosening (68.2%), mechanical implant failure (11.4%), non-union following periprosthetic fracture (11.4%), polyethylene wear with large osteolysis (6.8%) and instability (2.3%). Regarding the explanted implants, in trauma patients 69.4% had a bicondylar TKA, 22.4% a constrained TKA, in 8.2% only the femoral component of a constrained TKA was revised to DFR and 2.0% had already a mega-prosthesis. Prior to elective DFR, 27.3% had a bicondylar TKA, 36.4% a constrained TKA, in 18.2% only the femoral component was revised and 15.9% had a mega-prosthesis, p = 0.003.

**Table 3:** Results of the multivariate Cox regression model: factors associated with risk for revision of DFR.

Variable	Ref.	HR	95% CI	p-Value
age		0.981	(0.950; 1.013)	0.244
gender (female)	male	**0.379**	**(0.164; 0.874)**	**0.023**
elective DFR	trauma DFR	**3.021**	**(1.210; 7.541)**	**0.018**
explant (constrained)	(non-constrained)	**5.771**	**(1.089; 30.589)**	**0.039**
number of surgeries	1	0.521	(0.099; 2.751)	0.062

Revision rates in the both groups were 22% and 41%, respectively. Infection of DFR was the leading cause of revision in both groups (10% in trauma and 18% in elective patients).

**Fig 5:**
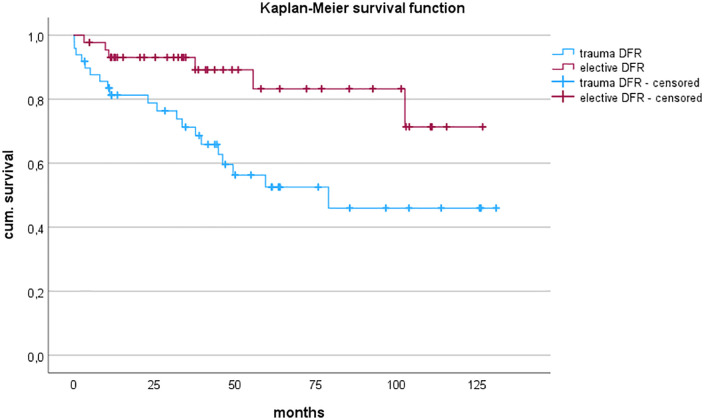
Kaplan-Meier-curve (patients survival).

The 1- and 5-year mortality rates were 18.7% and 47.5% for trauma patients and 6.9% and 16.8% for elective patients, see Fig 5.

Mean Satisfaction was numerically higher in trauma patients (6.9/10) compared to elective patients (5.4/10), but this difference was not statistically significant (p = 0.165). Mobility was limited, with 33% of trauma and 13.6% of elective patients unable to walk. Additionally, 71.4% of trauma and 45.5% of elective patients used a walking frame or wheelchair, and 52.4% of trauma and 22.7% of elective patients could not climb stairs.

## Discussion

Distal femoral replacement is a complex procedure often used as a last option in both, trauma and elective cases. In this study trauma patients had higher mortality rates but fewer revision surgeries compared to elective patients. Trauma and elective patients differed significantly in pre-existing conditions before DFR, prior surgeries, size and constraint of implant and age. The differences were therefore probably caused by these pre-existing conditions.

We have seen the typical geriatric patients with a mean age of 79 and 72 years in trauma and elective patients and predominantly woman, especially in trauma patients with 74% vs. 59% in elective patients.

**Revision rate** after one year was low in both cohorts (4.1% and 7.0%). After five years we found a relevant difference in revision rates of 13.9% in trauma and 42.0% in elective patients. This observation is supported by the multivariable Cox regression analysis, which demonstrated a significantly higher revision risk for elective DFR compared with trauma indications. This may be explained by the more difficult situation in elective cases due to larger implants and higher numbers of previous revisions and lower mortality in this group. There are a few single-center studies about long-term results in DFR, reporting between 10.2% to 60% revision rates at five and ten years [17–19]. Wyles et al. showed an increased risk of re-operation in patients who underwent index DFR for aseptic TKA loosening (HR 2.3; p = 0.026) or periprosthetic joint infection (HR 2.18; p = 0.022) compared to periprosthetic or native femoral fractures up to 10 years follow-up [18]. Even higher revision rates were reported for trauma DFR by a registry study with 32.7% after four years, whereas periprosthetic joint infection (PJI) with 12.8% was the most common reason for revision [20]. Infection rate leading to revision in DFR was 10% in this cohort.

Patients in our study who were men and who had already constrained TKA were at higher risk for revision to DFR. Higher risk of revision for aseptic loosening (but no higher risk for all-cause revision) in men has already been reported [18]. This study reported no explanation for the higher risk in men. We assume, that there might be higher mobility and excessive load in men compared to women.

**Mortality** was higher in trauma patients and comparable to reported mortality rates of 43% after five years in another single-center analysis [21] or in a register-based study with 48.2% after five years [12]. Mortality in patients with periprosthetic femur fracture seems to be independent of the surgical procedure. A systematic review about periprosthetic distal femur fractures compared different surgical interventions and reported 13% mortality rate after mean follow-up of 2.5 years for DFR compared to 13% for ORIF or 13% for intramedullary nail [5].

Patients risk for mortality is well known and substantial efforts and legal requirements are introduced to optimize the management in elderly patients with proximal femur fractures. Especially modifiable treatment factors as well as interdisciplinary treatment were implemented in order to reduce complications and mortality [22,23]. Patients with DFR following trauma are at the same risk and similar perioperative management programs should be introduced to improve the outcome.

**Function**: In this study almost all patients were reduced in their mobility, especially trauma DFR. In another single-center study about 52 patients with elective DFR, 60% of the patients needed mobility aids at mean follow-up of 3.5 years [24]. These findings confirm the vulnerability and frailty, disability and need for long-term care in these patients. However, the possibility of immediate postoperative mobilization with unrestricted weight bearing is often one crucial argument for DFR instead of fixation [13,25,26]. Reduced function in DFR after failed TKA has been reported in a single-center study, in which patients after primary TKA achieved a Knee Society function score of 90.17 ± 7.25, while patients after DFR achieved 23.1% of Knee Society function score compared to patients with primary TKA. (p < 0.001) [17]. This finding underlines the impaired function following multiple surgeries and the primary aim of a leg-saving salvage procedure in such cases. Although satisfaction tended to be higher after trauma, this difference did not reach statistical significance and should therefore be interpreted cautiously.

### Limitation and strengths

The actual cohort size was smaller than the estimated requirement based on published rates, and therefore the statistical power to detect between-group differences is limited. Therefore, comparisons between trauma and elective patients may be underpowered, and non-significant findings should be interpreted with caution. In addition, the number of revision events was relatively small, which may limit the stability of the multivariable regression model and therefore requires cautious interpretation of these findings. Nevertheless, the strength of this study is its sample size, which is one of the largest published single-center cohort as well as the detailed data of these special patient cohort. Information about revisions and mortality were available in nearly all patients. Nevertheless, we acknowledge further limitations of this study. Comorbidity was assessed only by the ASA classification, which does not capture specific diseases such as malignancy or cardiovascular disorders; thus, the overall comorbidity burden and its impact on mortality may have been underestimated. The majority of patients had their previous surgeries in other hospitals and data about initial indication criteria in primary as well as first revision surgery are often missing. Functional and satisfaction data were available in less than half of the cohort due to deaths, revisions, or loss to follow-up. As missing data were not imputed, this may have led to selection bias towards healthier survivors, limiting the generalizability of the functional outcome results. The wide range of follow-up times (1–10 years) represents a limitation of the study. As functional capacity and mobility tend to decline with age, later follow-up assessments may underestimate satisfaction and activity compared to earlier evaluations. Consequently, differences in timing may have introduced variability particulary in the satisfaction scores, and should be considered when interpreting these results

## Conclusions

DFR was associated with high revision rates, especially in elective cases, indicating it should be used cautiously. Trauma patients, despite fewer prior surgeries, faced higher mortality rates, underscoring the need for careful patients management and consideration of individual patient conditions.

## Supporting information

S1 File Study protocol followup knee revision UKD 2011 EN. (PDF)

S2 File TREND DRF 11.03.2025. (DOCX)
