# Supplementary material for: Outcome of distal femoral replacement: Influence of trauma vs. elective indications – A prospective single-center study
Source: PLoS One. 2026 Jul 24;21(7):e0354235. doi: 10.1371/journal.pone.0354235 (PMC13399309; doi:10.1371/journal.pone.0354235)
Supplement: S1 File — (PDF) [file pone.0354235.s001.pdf]

Study Protocol

Follow-up of patients in the study “Prospective single center study of knee arthroplasty revision surgeries”

EK 348112009

Study Center

Orthopedic Department
University Hospital Carl Gustav Carus Dresden
Director: Prof. Dr. med. K.-P. Günther
Medical Director

Senior Physician

Dr. med. Jörg Lützner

Dresden, 14.07.2011

Inhalt

1. Synopsis 3
   1.1. Background 3
   1.2. Study process 3
   1.3. Analysis strategy 4
2. Methodology of the Study 4
   2.1 Patient Recruitment 4
   2.2 Timeline 5
   2.3 Data Collection Instruments 5
   2.4 Data Management and Data Protection 6
   2.5 Data Analysis 7
   2.6 Software 7

1. Synopsis

1.1. Background

Knee arthroplasty revisions are associated with considerable costs, and outcomes are generally less favorable compared to primary arthroplasties, making them highly relevant from a socioeconomic perspective.

From the patient's perspective, the individually expected prognosis is relevant, which entails a residual risk of complications or adverse outcomes. This must be weighed against the expected (functional) benefit and appropriately communicated to the patient during preoperative counseling. In contrast to primary knee arthroplasty, there is a lack of data for knee revision surgeries in this regard. Therefore, the knee arthroplasty revisions currently being prospectively documented at the Department of Orthopaedics at the University Hospital Dresden (EK348112009) will be followed up long-term.

1.2. Study process

Patients who participated in the study “Prospective documentation of knee arthroplasty revision surgeries” (EK 348112009) will continue to be followed up at 3, 5, and 10 years postoperatively. These follow-ups will take place within the framework of routine postoperative care following knee revision surgery.

The following parameters will be collected:

- Activity: UCLA Activity Score

- Quality of life: SF36

- Function: Knee Society Score

1.3. Analysis strategy

Clinical outcome endpoints are the quality of life and functional scores at each follow-up. Stratification based on the causes leading to revision will be performed.

Values will be compared with previous data from our own studies on primary arthroplasty and with the literature.

The study aims to clarify the following questions:

1. What long-term clinical outcomes can be achieved after revision surgery?

2. Are these outcomes dependent on the causes that led to revision?

2. Methodology of the Study

2.1 Patient Recruitment

All patients who participated in the primary study "Prospective Collection of Knee Arthroplasty Revision Procedures" (EK 348112009) are planned to be included in this follow-up study.

During the 1-year follow-up (or via written/telephone contact if already completed), patients will be informed verbally and in writing about the further follow-up examinations, including their purpose and content.

After providing written consent for participation and data protection, patients will be invited for routine follow-up visits at 3, 5, and 10 years postoperatively. Patients will complete the questionnaires independently, with a documentation assistant from the Clinical Epidemiology division available to answer questions.

The completed documentation forms will be archived in the Clinical Epidemiology division after completion by the patients.

2.2 Timeline

Time Point	EK 348112009	1 year postop	3 years postop	5 years postop	10 years postop
Clinical Examination (Knee Arthroplasty Clinic)	X	X	X	X	
UCLA Activity Score	X	X	X	X	
SF-36	X	X	X	X	
Knee Society Score	X	X	X	X	
AE / SAE	X	X	X	X	

2.3 Data Collection Instruments

Most of the patient data will be collected using standardized questionnaires:

- **UCLA Activity Score, Knee Society Score, SF-36**.

  Data from the clinical examination will be taken from the records of the arthroplasty outpatient clinic.

The documentation forms will only contain a sequential patient number, which links the follow-up questionnaires with previously collected data. This number will also be recorded in the patient's medical record, allowing plausibility checks before statistical analysis if necessary. The data will then be transferred to a database.

In this database, preoperative, intraoperative, and follow-up data will be linked to the patient number while ensuring that patient identification by third parties is

excluded, thereby meeting the data protection requirements under German Social Code (SGB V).

Collected data includes:

1. **UCLA Activity Score** (self-assessment, 1 = completely inactive, 10 = regular participation in demanding sports).
2. **SF-36 Quality of Life Questionnaire**, German version (validated for practicability, validity, and reliability).
3. **Knee Society Score** (split into Knee Score and Function Score, each with a max score of 100; Knee Score includes pain, mobility, stability, and alignment; Function Score includes activities of daily living such as walking, stair climbing, and use of walking aids).
4. **Additional clinical data** from patient records and standard pre- and postoperative diagnostics:
    - Limb alignment
    - Position and potential loosening of prosthetic components

2.4 Data Management and Data Protection

Data sets will be managed and archived in a master database in an anonymized form. The sequential patient number allows clear allocation to the medical record if plausibility checks are required, but access is restricted to the Clinical Epidemiology staff and only upon explicit request by the treating physician. The database will not contain names or identifiers, ensuring third-party access is impossible.

Data will only be analyzed and published in anonymized, aggregated form (e.g., medians). Patients will be informed of this during the consent process and will provide written consent for data use and protection.

2.5 Data Analysis

Clinical outcome endpoints will be the score values for quality of life (SF-36) and function (Knee Society Score) at the respective follow-up points. Stratification based on the causes leading to revision will be conducted. Values will be compared with our previous data on primary arthroplasty and literature data.

2.6 Software

The patient-completed forms will be entered into an EXCEL® spreadsheet and transferred to an SPSS® database, where an initial plausibility check will be performed. All analyses will be conducted using SPSS® by the Clinical Epidemiology staff. At no point will third parties have access to electronic or paper documentation.

Dresden, 07-14-2011

Director, Department of Orthopaedics, Head of Arthroplasty Division)	Senior Physician, Study Medical Supervisor	Senior Physician, Arthroplasty Division
